# Impact of lactoferrin on bone regenerative processes and its possible implementation in oral surgery – a systematic review of novel studies with metanalysis and metaregression

**DOI:** 10.1186/s12903-020-01211-6

**Published:** 2020-08-26

**Authors:** Grzegorz Trybek, Maciej Jedliński, Aleksandra Jaroń, Olga Preuss, Marta Mazur, Anna Grzywacz

**Affiliations:** 1 Department of Oral Surgery, Pomeranian Medical University in Szczecin, al. Powstańców Wielkopolskich 72/18, 70-111 Szczecin, Poland; 2 Department of Oral and Maxillo Facial Sciences, Sapienza University of Rome, Via Caserta 6, 00161 Rome, Italy; 3 Student Scientific Society at the Department of Oral Surgery, Pomeranian Medical University in Szczecin, al. Powstańców Wielkopolskich 72/18, 70-111 Szczecin, Poland; 4 Independent Laboratory of Health Promotion, Pomeranian Medical University in Szczecin, ul. Gen. Dezyderego Chłapowskiego 11, 70-103 Szczecin, Poland

**Keywords:** Lactoferrin, Bone regeneration, Bone remodeling, Dental implants, Animal study, Oral surgery

## Abstract

**Background:**

Lactoferrin is an iron – binding glycoprotein with anti-inflammatory and anabolic properties found in many internal fluids. It is worth looking at novel studies, because of their methodology and observations that may once be applicable in modern implantology.

The aim of the study is to answer the question if lactoferrin is a promising factor for bone regenerative process in oral surgery.

**Method:**

An electronic search was conducted on 14th October 2019 on the PubMed, Scopus and Web of Science databases. The keywords used in the search strategy were: lactoferrin AND bone regeneration AND oral surgery. The qualitative evaluation was conducted using the Jadad and Newcastle-Ottawa Quality Assessment Form. Then a metanalysis of a new bone growth and percentage of the resorbed graft were performed with the metaregression of lactoferrin dose to its outcome effects on bone regeneration.

**Results:**

The search strategy identified potential articles: 133 from PubMed, 2 from Scopus, 4 from Web of science. After removal of duplicates, 136 articles were analyzed. Subsequently, 131 papers were excluded because they did not meet the inclusion criteria. The remaining 5 papers were included in the qualitative synthesis. The use of lactoferrin clearly increases the growth of a newly formed bone (2.58, CI:[0.79, 4.37]), as well as shortens the time of the graft resorption (− 1.70, Cl:[3.43, 0.03]) and replaces it with a species-specific bone. Heterogeneity is significant at *p* < 0.001 level. Metaregression indicates that one unit increase in the log (Treatment dose), i.e. a 2.78 times increase of the Treatment dose, results in an increase of the Effect size by 0.682.

**Conclusions:**

The use of lactoferrin both systemically and locally promotes anabolic processes (new bone formation). There is a relationship between the increase in administered dose of lactoferrin and the intensity of new bone formation. However, it is not only necessary to continue experimental research, but also to extend it to the clinical studies on patients, due to the limitations of different animal model research and different methodology, to introduce lactoferrin as a standard procedure for the treatment of bone defects, because it is a promising product.

## Background

Lactoferrin is glycoprotein 80 kDa able to chelate two ferric ions per molecule. It is a component of many externally secreted substances such as saliva, tears, colostrum, milk, gastrointestinal fluids, nasal and bronchial mucosa [1]. Lactoferrin contains 691 amino acid residues assembled into two homologous lobes connected through a peptide. All these structures form a 3-turn α-helix. This glycoprotein is able to retain iron ions in the chelated form to pH values as low as 3.0. Because of its structure, it is a component of the innate immune response and a potent immunomodulator [2, 3]. Its ability to bind free iron ions as well as deactivate reactive oxygen forms prevents the tissues from excessive inflammatory processes and also decreases bacterial growth and the development of biofilm [2, 4].

It was also found to have a direct effect on cell differentiation and growth [5], as well as modulation of the cytokine production processes i.e. the ones with direct impact on bone tissue regeneration and growth [6–8]. Lactoferrin stimulates the growth and activity of chondrocytes and osteoblasts, while inhibiting osteoclastogenesis, without affecting mature osteoclast activity [9]. Its administration decreases secretion of a number of cytokines such as tumor necrosis factor α (TNFα) and interleukin 1 beta (IL-1β) which promote osteolytic processes in tissues [7, 8], and at the same time stabilize the balance in the Receptor activator of nuclear factor κ B (RANK)/ Receptor activator of nuclear factor κ B Ligand (RANKL)/ osteoprotegrin (OPG) system [9]. Since the discovery of the RANKL / RANK / OPG system in the mid-1990s, there is a much more considerable understanding of osteoclast formation and activation. Osteoblasts and stromal stem cells express the receptor activator of ligand (RANKL), which binds to its receptor, RANK, on the surface of osteoclasts and their precursors which activate them. If the ligand is present, this system increases the differentiation of precursors into multinucleated osteoclasts and the activation of osteoclasts and survival in both physiological and pathological conditions associated with increased bone resorption. When the ligand is no ligand, the differentiation of osteoclast is decreased. Osteoprotegrin (OPG) is secreted by osteoblasts and osteogenic stromal stem cells and protects the skeleton from excessive bone resorption by binding to RANKL and preventing its interaction with RANK [10]. Lactoferrin stabilizes the osteinductive effect of hydroxyapatite crystals by acting on osteoblast and osteoclast cells through the RANK / RANK / OPG system mentioned above [11].

In 2006 it was discovered how lactoferrin acts by directly reinforcing bone healing processes at the cellular level [12]. Lactoferrin has a mitogenic effect on osteoblasts close to the wound, mediating through the low-density lipoprotein receptor-related protein-1 (LRP1) and activating two kinases of osteoblast cells: p42/44 mitogen-activated protein kinase (MAPK) and the PI3-kinase-dependentphosphorylation of Akt [12]. Recently, lactoferrin has become the focus of many studies. This increasing interest may result from a deeper understanding of its function and from its greater availability to scientists and practitioners. This results from the fact that the species of origin and the method of preparation of lactoferrin does not substantially affect the extent of proliferation on osteoblast cells [8], which makes the studies on lactoferrin and its possible implementation in medicine cheaper and more universal.

## Aim of the study

The aim of the study is to answer the question if lactoferrin is a promising factor for bone regenerative process in oral surgery.

## Method

### Search strategy

**Fig. 1:**
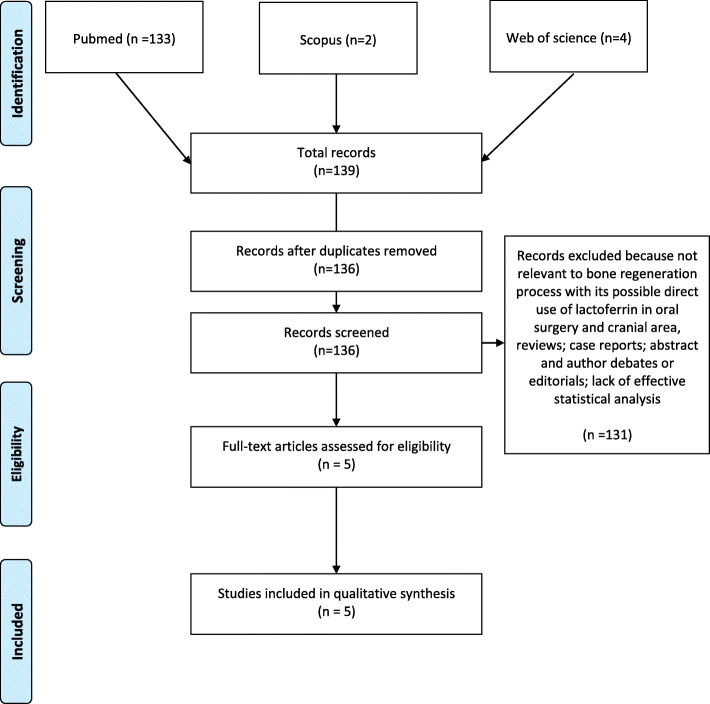
Primary 2009 Flow Diagram

This review was performed under the PRISMA guidelines [13]. The results are presented in and Fig. 1. An electronic search was conducted on 14th October 2019 on the PubMed, Scopus and Web of Science databases. All searches were conducted using a combination of subject headings and free-text terms: we determined the final search strategy through several pre-searches. The keywords used int the search strategy were: lactoferrin AND bone regeneration AND oral surgery. Reference lists of primary research reports were cross-checked in an attempt to identify additional studies.

### Eligibility criteria

The following inclusion criteria were employed for this systematic review: (1) randomized clinical trial; (2) cohort study; (3) case-control study; (4) articles published in the last 10 years; (5) studies carried out on human subjects; animal subjects (6) results published in English (7) studies on lactoferrin impact on bone tissues.

The following were the exclusion criteria: (1) reviews; (2) case reports (3) abstract and author debates or editorials; (4) studies not related to bone regeneration; (5) papers not related to possible direct use of lactoferrin in oral surgery; (6) lack of effective statistical analysis.

### Data extraction

First, two reviewers selected the studies by reading titles and abstracts and then by studying the full texts of select articles (MJ and OP). Any doubt or disagreement between the two reviewers was resolved by discussion with a third author (AJ). All data extracted from the selected studies are shown in the Table 1.

**Table 1:** Data extraction and characteristics of studies included in review

Author and year of publication	Type of study	Bone structure under examination	Subject type	Number of subjects	Follow-up	Test group	Control group	Method of lactoferrin supply	Results
**Yoshimaki T. et al. (2014)** [14]	Case – control study	Cranium, non-critical-sized calvarial defects in the parietal area	Fischer rats	10 – in every rat 2 trepanations - one on each side of midsagittal suture	4 weeks CT observ. and histopat. at the of study.	3 mm collagen membrane containing 11 μl of bovine LF solution (500 mg/ml, total 5.5 mg of LF) applied intraoperatively on defect	3 mm collagen membrane permeated with 11μl of saline solution applied intraoperatively on defect	Local administration	Newly generated bone was observed at LF sites as early as 2 weeks after surgery, but the significant differences between test and control group occurred at 3^rd^ and 4^th^ week of CT observation (1.70 vs. 1.13 mm^3^ and 2,44 vs. 1.54 mm^3^). The mass of new bone at sites with LF increased significantly at 4^th^ week 1.55 vs. 1.02 mg. In histopathological examination the number of pixel covered by new bone tissue was 2 times bigger than in control site, with almost 2,5 times bigger number of osteoblast-like cells.
**Yoshimaki T et al. (2013)** [15]	RCCT	Cranium, non-critical-sized calvarial defects in the parietal area	Fischer rats	30 – 10 in every group	4 weeks, CT observ. and histopat. at the of study.	1. Group - 100 mg LF/kg + Collagen membrane 2. Group – 10 mg LF/kg + collagen membrane intraperitoneally injection every day, starting on the day of the operation	Collagen membrane + saline injections, intraperitoneally injection every day, starting on the day of the operation	Systemic administration	Newly generated bone was observed during CT observation 2 weeks after surgery in the LF groups, with only minimal formation of new bone in control group. The significant differences between test and control group occurred at 3^rd^ and 4^th^ week of study. In both test groups the collagen tissue was absorbed. In test group the fibrous tissue with osteoblasts and new bone covered the bone sites, many times more prominent in the 100 mg LF/kg. The control was filled with fibrous connective tissue; with minimal regeneration sites on defect rims. There was no Ca ions and ALP concentration differences in serum between two groups.
**Görmez et al. (2015)** [16]	Case – control study	Cranium, bone defects in frontal bone area	Domestic pigs	24 uniform bone defects in 12 pigs	10 weeks, no intervention during the follow-up	Placement of a titanium implant + 0.3 mL inorganic bovine-derived graft + bovine lactoferrin loaded gelatin microspheres + collagen membrane (Lactoferrin group)	1. Placement of a titanium implant + 0.3 mL inorganic bovine-derived graft + Collagen membrane; (Graft group) 2. Placement of a titanium implant + Collagen membrane; (Empty defect group)	Local administration	After 10 weeks of uneventful healing, all the implants showed successful osseointegration. Different amounts of newly formed bone defects were seen in all treatment groups. In the bovine lactoferrin group, significantly more bones around the implants were observed and bone growth above the tip of the implants was detected. Lost tissue regeneration amounted 26.9 ± 6.0% in the empty defect group, 31.8 ± 8.4% in the graft group, and 47.6± 5.0% in the lactoferrin group. In the group in which lactoferrin was used, most of the graphite was resorbed and replaced with subjects own bone.
**Paknejad et al. (2013)** [17]	RCCT	Cranium, non-critical-sized calvarial defects in the parietal area	Male New Zealand rabbits	32 bone defects in 8 rabbits	4 weeks	Inorganic bovine-derived graft (Bio oss)+ Non-iron saturated bovine lactoferrin + Tragacanth (carrier) + collagen membrane	1. Inorganic bovine-derived graft only 2. Inorganic bovine-derived graft + Tragacanth only 3. Inorganic bovine-derived graft + Lactoferrin only	Local administration	All groups were not scientifically different in terms of inflammation, vitality and percentage of new bone formation nor residual Bio oss material. The percentage of new bone formation in test group was 15.95 ± 2.24%, while in control groups were 1^st^. - 13.44 ± 2.89%; 2^nd^. - 14.73± 3.14%, and 3^rd^.-15.02 ± 1.51%. Although the mean values differ from each other, the standard deviation in all groups makes the results statistically insignificant. The results of this study are in contradiction to others investigating the effect of lactoferrin on bone regeneration rapitidy within the skull.
**Gao et al. (2018)** [18]	RCCT	Cranium, critical-sized calvarial defects in the parietal area	Sprague–Dawley rats	One 5mm critical-sized defect in each of 64 subjects	4 weeks and 12 weeks	Lactoferrin + collagen gel (10 μg/gel)	1. No intervention 2. Collagen gel only 3. Sham surgeries (skin and periosteal incisions only)	Local administration	The bone volume fraction (BV/TV) was higher in lactoferrin-treated animals at both timepoints compared to the other groups with critical-sized defects, 16.5 ± 0.6% (4 weeks) and 21.9 ± 1.2% (12 weeks), while only 10.5 ± 1.1% and 12.2 ± 1.3% in the group without any intervention. The animals in group 4 (sham surgeries) had the highest percentage of newly formed bone compared with the other three groups at both 4 weeks (24.27 ± 2.6%) and 12 weeks (29.3 ± 0.8%). The lactoferrin treated group had greater amounts of regenerated bone at both 4 and 12 weeks. A mixture of lamellar and woven bone was observed in all critical-sized defects groups throughout the study.

*RCCT* randomized controlled clinical trial, *LF* lactoferrin, CT computed tomography

### Quality assessment

The quality assessment was performed using the Jadad scale for reporting randomized controlled trials for RCT and RCCT studies [19]. The results are shown in Table 2. In assessment it was taken into account if the study was randomized and double-blinded with appropriately described methods. For every characteristic evaluated a point was given. Assessment ranged from zero to five with a high score indicating a good quality of study. Notwithstanding, for Case-control Studies the Newcastle-Ottawa Quality Assessment Form [20] was used. The results are shown in Table 3. The qualities of all included case-control studies were based on object selection, comparability, and exposure. The possible quality assessment score ranged from zero to nine points with a high score indicating a good quality study. For each characteristic evaluated one point was given. To investigate the risk of a publication bias, our search was conducted on www.controlled-trials.com and www.clinicaltrials.gov to verify the number of ongoing studies in this field. No such studies were found.

**Table 2:** Scoring according to Jadad scale for reporting randomized controlled trials [19]

Author	Yoshimaki T. et al. (2014) [14]	Paknejad et al. (2013) [17]	Gao et al. (2018) [18]
Randomization present	1	1	1
Appropriate randomization used	1	1	1
Blinding present	0	1	1
Appropriate blinding used	0	0 – not described	0 – not described
Appropriate long-term follow-up for all patients	1	1	1
Total	3	4	4

**Table 3:** Scoring according to Newcastle-Ottawa Quality Assessment Form for Case-control Studies [20]

Study	Terms	Görmez et al. (2015) [16]	Yoshimaki T. et al. (2013) [14]
Selection	Is the case definition adequate?	1	1
	Representativeness of the cases	1	1
	Selection of Controls	1	1
	Definition of Controls	1	1
Comparability	Comparability of cases and controls on the basis of the design or analysis	2	2
		Both test and control surgical sites underwent the same procedure at the begging of the study. The race, age and the breeding place in every group were the same. Confidence in comparability of results is contributed by the fact that both test and control sites were in the same individuals.	Both test and control groups underwent the same procedure at the begging of the study. The race, age and the breeding place in both groups were the same. Authors maintain, that all the subjects got the same number of injections at a similar frequency.
Outcome	Ascertainment of exposure	1	0 – not described
	Same method of ascertainment for cases and controls	1	0 – not described
	Non-Response rate	No description	No description
Total		8	6

### Summary measures and heterogeneity

A percentage of bone regeneration and differences in the residual amount of the graft were taken as a measure of treatment efficacy for both groups – Treatment group (TG) and Control group (CG). Meta-analysis was performed using the random-effect model via metafor and compute.es R packages, with Standardized Mean Differences (SMD) and 95% confidence intervals (95% CI) being calculated as effect estimates. Heterogeneity was assessed quantitatively using I2-statistics and Cochran’s Q [21]**.** In studies examining more than one factor affecting bone regeneration, the test and control groups were separated so that the only differentiating factor was the use of lactoferrin, or lack of it. There were 5 literature positions included in metaanalysis. Treatments with 10 and 100 mg/kg in Yoshimaki et al. [15] were treated as separate studies, as well as the cases with and without a carrier in Paknejad et al. [17] and 4 and 12 weeks measurements in Gao et al. [18] These resulted in 8 studies for bone regeneration and 3 studies for Differences in the residual graft percentage.

### Metaregression of a lactoferrin dose to its outcome effects on bone regeneration

To perform metaregression, only papers enabling full conversion of lactoferrin doses between studies were used (mg/kg.b.w). In order to determine if the treatment dose accounts for the dispersion in the summary effect, meta-regression model

$$ Effect\ size={\beta}_0+{\beta}_1\log \left( treatment\ dose\right) $$

was examined. The input data are presented in Table 4.

**Table 4:** Characteristics of Metaregression with the method of calculating the dose throughout the studies

	Effect size	Treatment dose (mg/kg.b.w)
Yoshimaki T. et al. (2014) [14]	1.19	20
Yoshimaki T et al. (2013) [15] 100mg/kg	2.02	100
Yoshimaki T et al. (2013) [15] 10mg/kg	0.62	10
Paknejad et al. (2013) [17] w/o carrier	0.40	10.77
Paknejad et al. (2013) [17] with carrier	0.46	10.77

## Results

### Search results

The search strategy identified potential articles: 133 from PubMed, 2 from Scopus, 4 from Web of science. After removal of duplicates, 136 articles were analyzed. Subsequently, 131 papers were excluded, because they did not meet the inclusion criteria. The remaining 5 papers were included in the qualitative synthesis (Flow diagram). Three of them are RCTs, while 2 of them are case-control studies. Table 1 summarizes the characteristics for each of the 5 studies included.

### Quality assessment and the risk of bias

None of the RCTs has received a maximum score on the Jadad Scale. This is due to the fact, that Yoshimaki et al. [20] did not use any type of blinding. Although Paknejad et al. [17] and Gao et al. [18] reported that the histological analysis was conducted by a blinded pathologist, there is no description of the blinding method in the article, hence a lack of certainty about its appropriateness. Because of the randomness of the way bone defects were filled during surgery in the same animal [15, 17] and full randomization of the selection of subjects in the Gao et al. study [18], without any doubts the randomization and the methods of its use were proper in all RCT studies. Case-control studies [14, 16] are characterized by their good methodological value. Both authors precisely described the test and control groups. Görmez et al. [16] assured the comparability of cases and controls by making similar bone defects in small group of animals of the same race from the same breed. The specimens were examined with the same equipment. On the other hand, in the Yoshimaki et al. [15] study, due to the method of lactoferrin supply (postoperative injections), a specific method of ascertainment of exposure was needed, which, unfortunately, was not described. Also, in this study all the specimens were examined with the same equipment [14]. Despite all of this, it should be pointed out that the limitations of the studies were included in this review. First of all, in all RCTs, even if it was reported that blinding was used, none of them described it thoroughly enough to determine if it was appropriate. It was practically in every study that a new bone tissue formation was observed. No consistent timepoint in the follow-up between them was found, which prevents us from a direct comparison of the test results and forces to compare them only by EF (effect size), which increases the risk of bias. Only - Görmez et al. [16] and Gao et al. [18] reported in their studies, that the temperature during drilling a defect was 37 °C and it was stable during the procedure, which is crucial for osteoblast survival in the immediate vicinity of the defect and therefore for a possible anabolic effect of lactoferrin on bone tissue. In 1983, Eriksson and Albrektsson proved irreversible histological changes in a rabbit’s tibia as a result of applying the temperature of 47 °C for more than 1 min. Even more damage occurred when the temperature rose to 53 °C. Overheating the bone tissue for more than 1 min at 60 °C causes complete interruption of the blood supply and tissue necrosis without the appearance of reconstruction exponents for up to 100 days of observation [22].

### Metanalysis

**Fig. 2:**
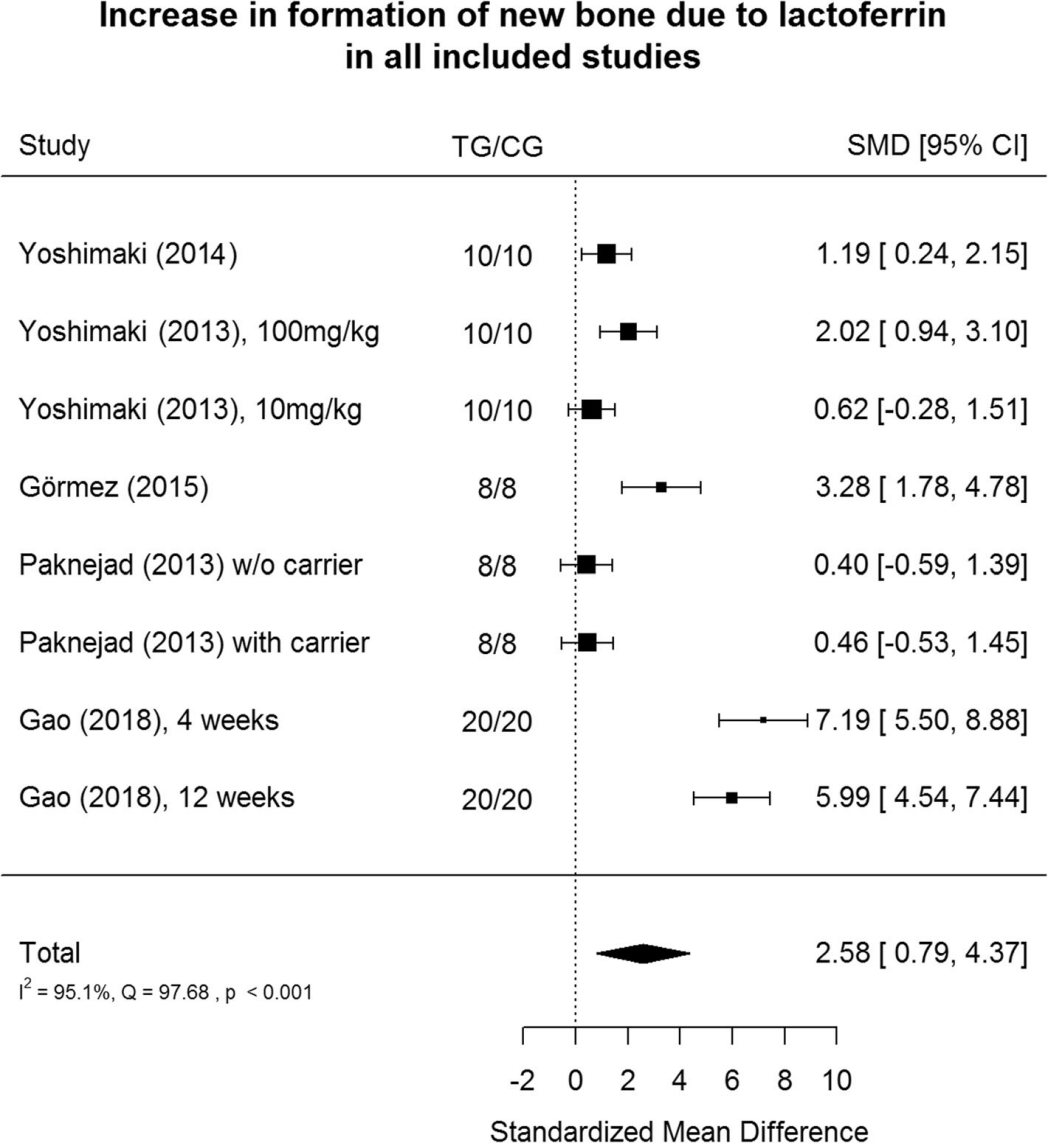
Metanalysis Tree Diagram - This diagram shows the acceleration of the healing effect relative to the control sample among all the study groups in the articles included in the review

The results are shown in Fig. 2. Positive values of SMD indicates greater efficacy in TG (lactoferrin usage), negative – greater efficacy in CG.

Lactoferrin usage appears to have a large positive effect size (2.58, CI:[0.79, 4.37]) on bone regeneration, and it is large accordingly to Cohen’s interpretive guidelines [23] on bone regeneration in relation to the control group. Heterogeneity is significant at *p* < 0.001 level. The results of available studies are very different. 95.1% of the variability comes from heterogeneity (variability in sizes of effects that result from true differences among the studies) [24]. Large part of heterogeneity is introduced by Görmez et al. [16] and Gao [18] in their studies. In the Görmez and Gao studies the healing process lasted longer than in most of the studies mentioned above. Additionally, in Gao study lactoferrin was applied in different gel form (supplementary to collagen gel) than in the rest of the studies.

**Fig. 3:**
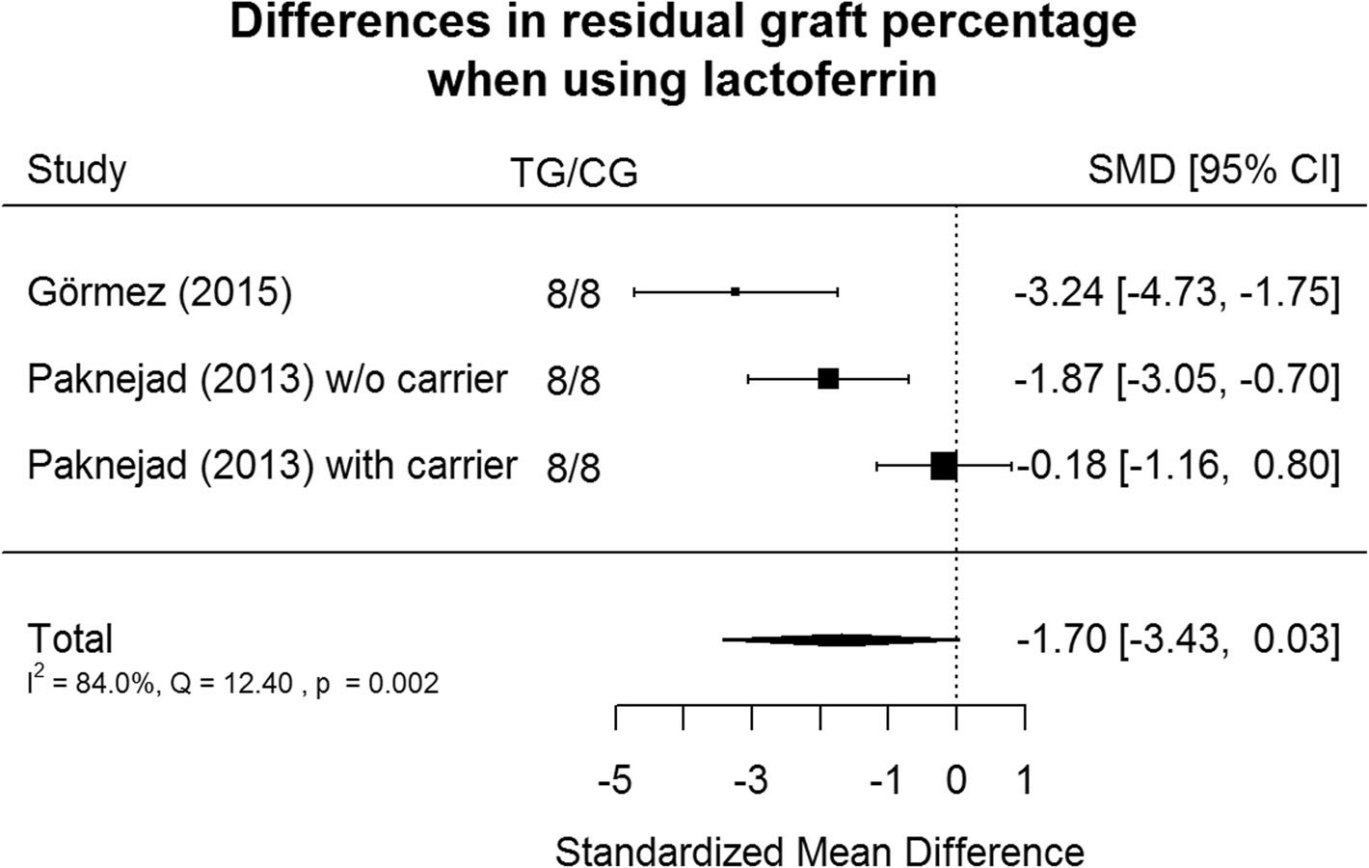
Metanalysis tree diagram - This diagram shows the acceleration of graft resorption relative to the control sample among all study groups in the articles included in the review. Along with the resorption of graft, it was replaced with host tissue

**Fig. 4:**
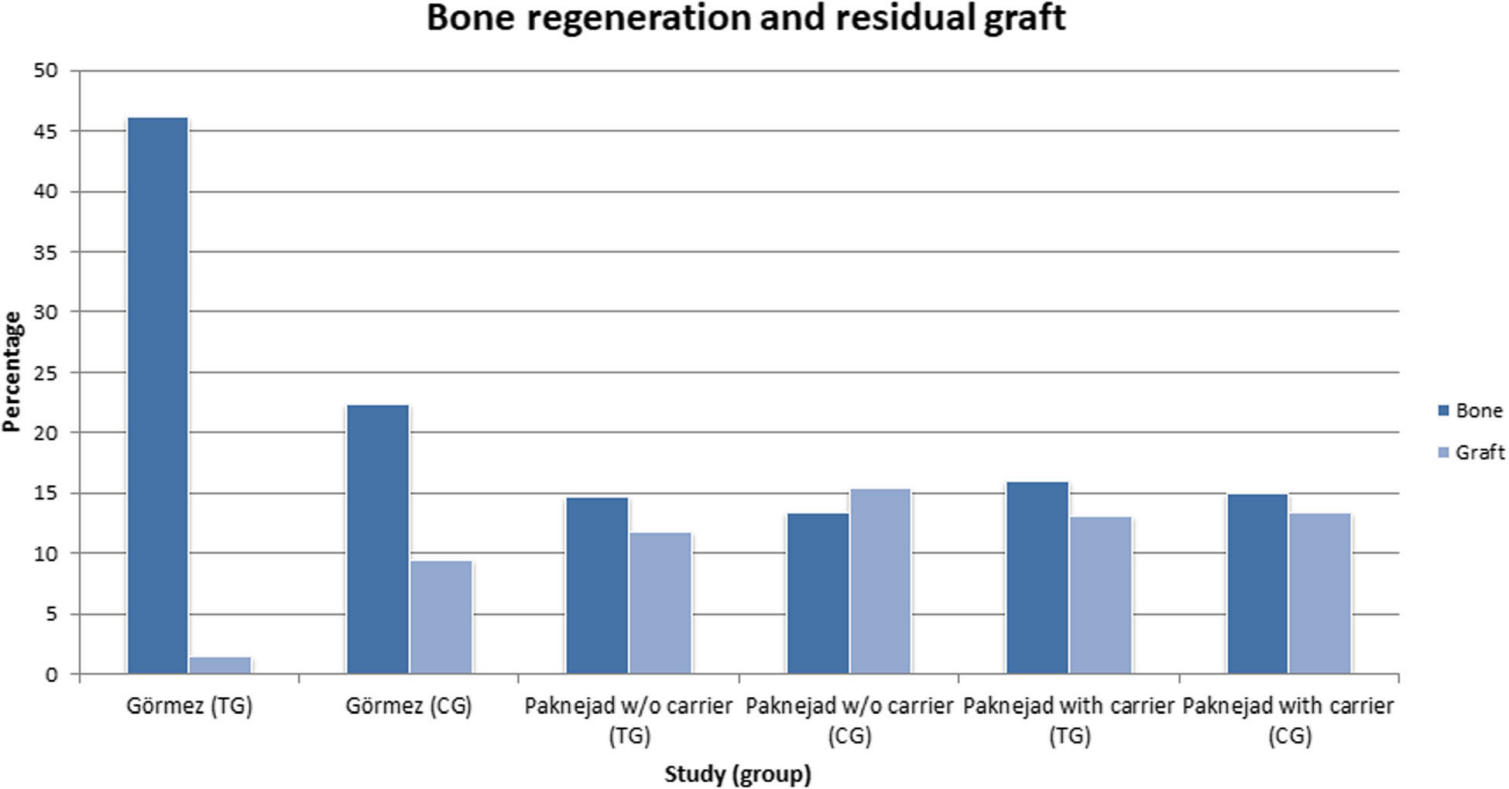
Column diagram, which is an overview of both effects throughout the studies

In each test group, faster graph resorption is noticeable (− 1.70, Cl:[3.43, 0.03]). The results are shown in Figs. 3 and 4.

It should be noted that a larger amount of the resorbed graft is always associated with a simultaneously greater increase in a newly formed bone, as a result of which the bovine bone is replaced by the species-specific tissue (newly formed bone), while maintaining the overall volume of bone tissue, which indicates stimulation of the regeneration process and undoubtedly should be considered as a successful treatment.

### Metaregression of a lactoferrin dose to its outcome effects on bone regeneration

**Fig. 5:**
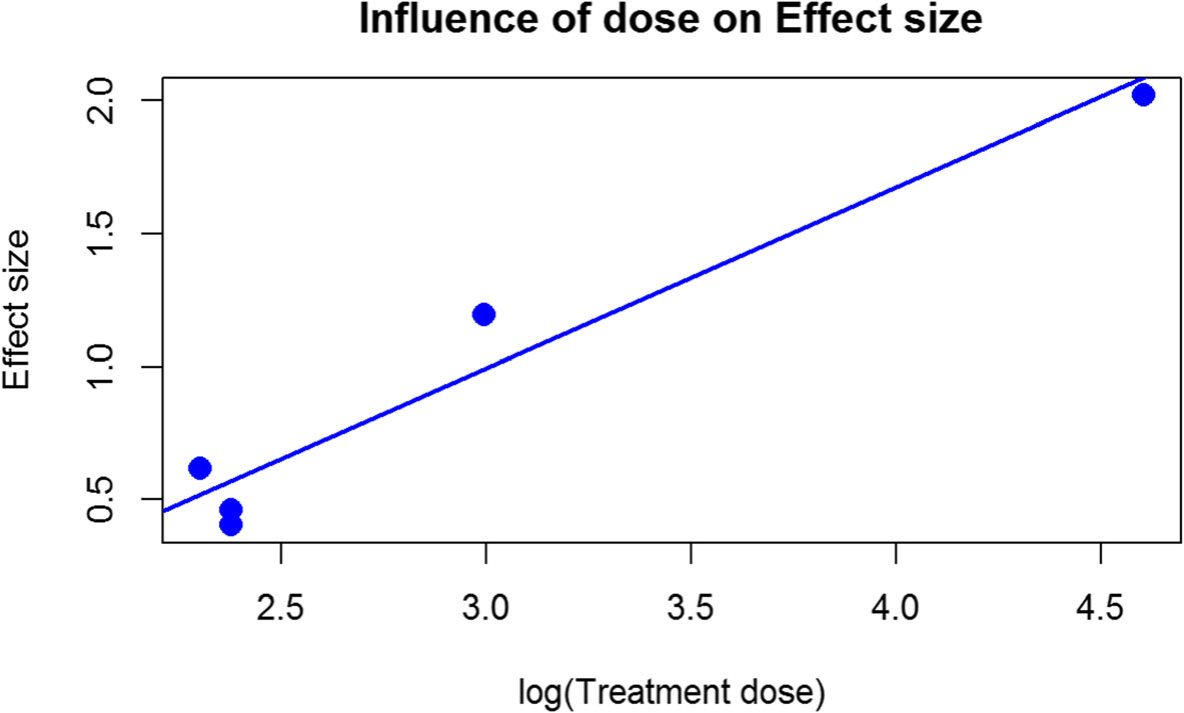
Diagram which indicates the dependence between increased dose of lactoferin and bone growth

**Fig. 6:**
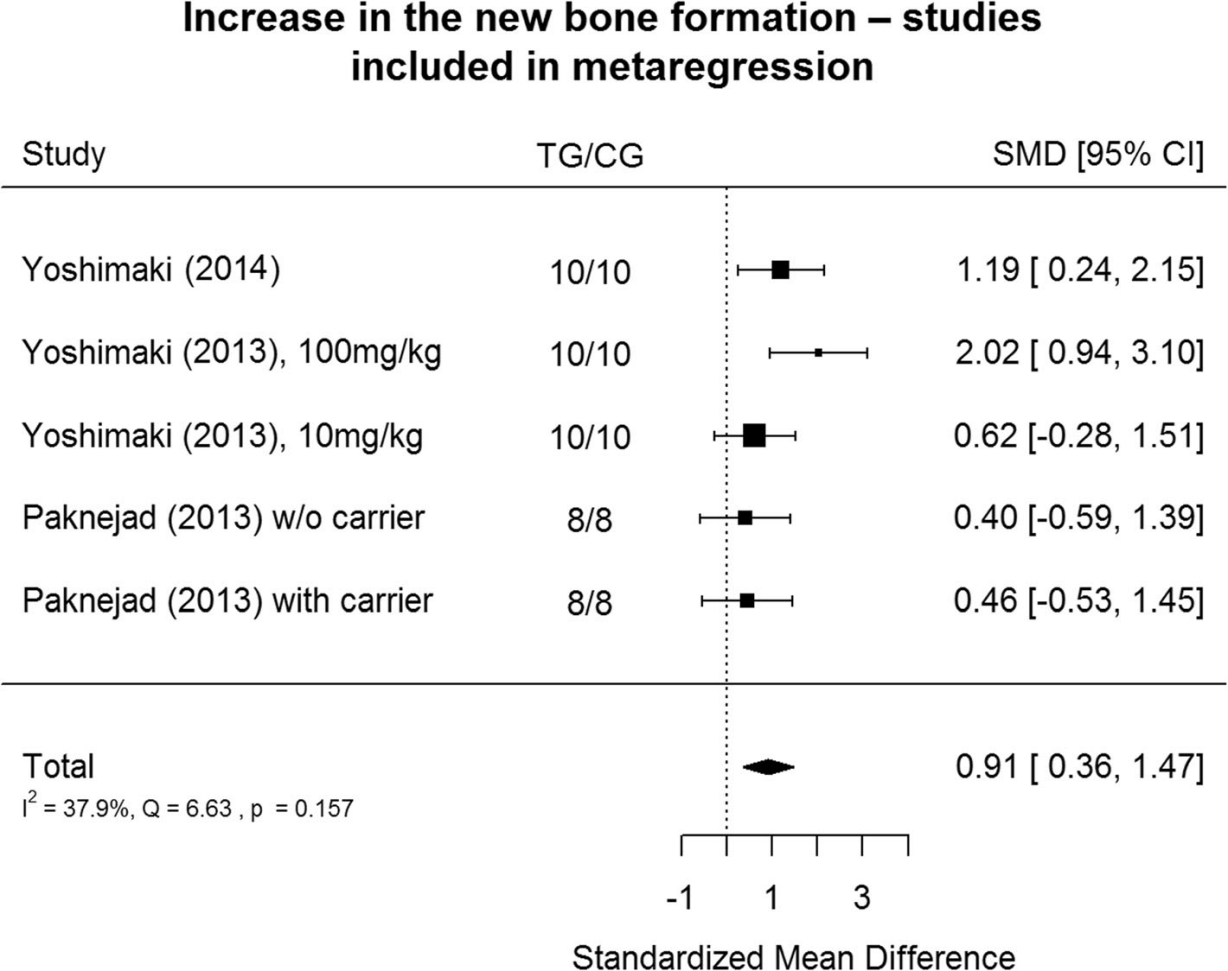
Metaregression tree diagram

Slope parameter β1 is statistically significant. One unit increase in the log (Treatment dose), i.e. a 2.78 times increase of the Treatment dose, results in an increase of the Effect size by 0.682. The treatment dose moderator accounts for a large proportion of the between-study variance, reducing I^2 estimate to zero, but has a high upper bound of confidence interval, so this result should be interpreted cautiously. However, they indicate a positive effect of increasing the dose on the percentage gain of a newly formed bone. The results of the model valuation are presented in Tables 4 and 5 and Figs. 5 and 6.

**Table 5:** Characteristics of Metaregression

Parameter	Estimate±SD	z	p
*β*_0_	−1.047 ± 0.810	−1.293	0.196
*β*_1_	0.681 ± 0.273	2.498	0.012
Heterogeneity			
*I*^2^	*I*^2^CI	Q	p
0.00%	[0.00, 44.64%]	0.338	0.943

### Summarizing findings

Our findings show inconsistent results on both measurements – bone regeneration and differences in residual. The bone regeneration treatment dose appears to be a good moderator for this inconsistency, at least when lactoferrin is applied locally. Unfortunately, there are too few studies to check if it is also the case for differences in the residual graft.

For all the studies the publication bias was assessed by analyzing funnel plots for differences in the residual graft as well as for bone regeneration. No significant bias of publications in the field of bone regeneration was detected in studies on smaller mammals (mice, rats, rabbits). Most of the degree of heterogeneity is caused by Görmez’s and Gao’s results.

## Discussion

As a stabilizer for the immune response to the inflammatory process lactoferrin has undoubtedly a visible effect on the process of bone formation. Many studies carried out in other parts of the body of experimental animals clearly indicate its positive effect [25, 26]**.** Recently it has been proved that the process of osteodistraction within rabbit’s fibula occurs more efficiently and has more stable results. Authors suggested that the increased tissue growth relative to the control group is attributed to the tilting balance in the RANK / RANKL / OPG system towards increased osteoprotegrin expression and significant decrease expression of RANK-ligand [11]. The same effect is indicated by the study carried out by Montesi et al. on cells culture [10].

In an organ culture study of intraverbal discs it was found that the addition of lactoferrin causes a number of not only anabolic but also anti-catabolic reactions. Through inhibiting the reaction sequence caused by Il-1 and lipopolisacharid (LPS) lactoferrin reduced the expression of multiple metaproteinaseas and disintegrin and metalloproteinase with thrombospondin motifs (ADAMTS) at the mRNA level, thus reducing tissue degeneration in bovine, rabbit and mouse cells in vivo and ex vivo [25]. Additionally, it increases several times the bone morphogenic protein 7 (BMP-7) gene expression relative to the control group. Furthermore, lactoferrin diminishes the presence of nitric oxide, which was associated as a pro-inflammatory factor, which leads to cartilage destruction in knee joints and spine discs [10, 11, 25]. Pelletier JP et al. indicted that the higher level of nitric oxide is directly proportional to a higher level of metalloproteinases (MMPs) [24]. The results of such studies are promising, because the human is one of the most biologically responsive species to bovine lactoferrin [27]. Several authors who also examined the degree of graphite resorption during a long-term follow-up found that the bovine graft (BioOss), used in all the studies, is gradually resorbed and replaced with the host bone with only a slight decrease in the bone volume [28]. Not only slow resorption [29], but also no resorption within six years [30] were reported in the literature. Among the studies reviewed in the review - Görmez et al. [16] found that the addition of lactoferrin during implantation significantly accelerates this process, even with a short (4 weeks) follow-up (− 3.42!). In research conducted by Paknejad et al. [17] the differences are also noticeable, but they are not so staggering. However, there is always the effect of speeding up the graft replacement process. With one exception (Yoshimaki et al.) [15], lactoferrin was administered locally in all evaluated studies. The question should be asked: Where does this tendency come from? During the implantation procedure, topical agents are already used to increase osteoblast proliferation, such as various growth factors, platelet-derived growth factor (PDGF) [31]**,** and accelerate their differentiation such as bone morphogenetic protein-2 [32]. Through its interaction with preosteoblasts lactoferrin provides an acceleration of both processes. Additionally, locally active lactoferrin increases the amount of growth factors, which can be combined with implants or orthopedic scaffolds to act synergistically in order to improve osseointegration and ultimately lead to better clinical results. What is more, systemic administrated lactoferrin is characterized by poor bioavailability in human subjects [33]. Free lactoferrin is a protein simply digested in the digestive system. The form of injection used by Yoshimaki et al. [14] overcame the problem of absorbability in the gastrointestinal tract, but it seems practically impossible to be introduced in a typical daily practice, whereas a local application as a possible alternative treatment is available. The possibility of using lactoferrin as a component of a dressing or collagen membranes during surgery saves the patient from an unpleasant series of injections and is much less time consuming. Nowadays, it should be stated that the therapeutic and utility potential of this glycoprotein should be considered untapped.

## Conclusions

Experiments using an animal model suggest that lactoferrin may be a useful factor in the regeneration of bone defect in the head and neck area. However, the purpose of our research was to determine whether it is a promising factor for bone regeneration in oral surgery. Nevertheless, it is not only necessary to continue experimental research, but also to extend it to the clinical studies on patients, due to the limitations of different animal model research and different methodology, to introduce lactoferrin as a standard procedure for the treatment of bone defects, because it is a promising product.

## Data Availability

Not applicable.

## Abbreviations

TNFα: Tumor necrosis factor α
IL-1β: Interleukin 1 beta
RANK: Receptor Activator for Nuclear Factor κ B
RANKL: Receptor Activator for Nuclear Factor κ B Ligand
OPG: Osteoprotegrin
LRP: Low-density lipoprotein receptor-related protein-1
MAPK: Mitogen-activated protein kinase
TG: Treatment group
CG: Control group
SMD: Standardized mean difernces
LPS: Lipopolischarid
ADAMTS: A disintegrin and metalloproteinase with thrombospondin motifs
BMP − 7: Bone morphogenic protein 7
MMPs: Metalloproteinases
PDGF: Platelet-derived growth factor
